# Comparison of Experienced and Inexperienced Raters Using Automated Deep Learning Computed Tomography Analysis to Evaluate Tricuspid Valve and Right Heart Morphology

**DOI:** 10.1016/j.shj.2025.100488

**Published:** 2025-05-06

**Authors:** Isabel Mattig, Maximilian Eichenberg, Elena Romero Dorta, Cheng-Ying Chiu, Nanike Bühring, Ann-Christine Stahl, Georg Böning, Lars-Arne Schaafs, Marc Dewey, Henryk Dreger

**Affiliations:** a Deutsches Herzzentrum der Charité, Department of Cardiology, Angiology and Intensive Care Medicine, Campus Charité Mitte, Berlin, Germany; b Charité – Universitätsmedizin Berlin, Corporate Member of Freie Universität Berlin and Humboldt-Universität zu Berlin, Berlin, Germany; c DZHK (German Centre for Cardiovascular Research), Partner Site, Berlin, Germany; d Berlin Institute of Health at Charité – Universitätsmedizin Berlin, BIH Biomedical Innovation Academy, Berlin, Germany; e Charité – Universitätsmedizin Berlin, Corporate Member of Freie Universität Berlin and Humboldt-Universität zu Berlin, Department of Radiology, Berlin, Germany; f Berlin University Alliance, Berlin, Germany; g Deutsches Herzzentrum der Charité, Department of Cardiology, Angiology and Intensive Care Medicine, Campus Virchow Klinikum, Berlin, Germany; h Structural Heart Interventions Program (SHIP), Deutsches Herzzentrum der Charité, Berlin, Germany

**Keywords:** Artificial intelligence, Computed tomography, Tricuspid regurgitation, Tricuspid valve intervention

## Abstract

• Computed tomography (CT) is used to plan tricuspid valve interventions.
• Deep learning-based CT analysis enables fast and accurate right heart assessment.
• Results are independent of the users’ experience.

In recent years, various interventional therapies, e.g., edge-to-edge therapies, percutaneous annuloplasty, or orthotopic valve implantation, have emerged to treat symptomatic, inoperable patients with tricuspid regurgitation (TR), as recommended by the current European Society of Cardiology guideline.^1^ The occurrence of TR increases with age, reaching a prevalence of 4% in patients older than 75 years.^2^ To plan interventional therapy for these patients—such as percutaneous annuloplasty or orthotopic valve implantation—computed tomography (CT) is routinely used to characterize tricuspid valve anatomy and right heart morphology.^3^ CT analysis based on artificial intelligence (AI) may simplify and accelerate the measurement process as well as enable a more cost-effective evaluation.^4^ However, a precise and ideally automated CT assessment is not yet established in clinical routine. Therefore, the present study aimed to evaluate new automated deep learning-based CT analysis software and its impact on measurements performed by experienced and inexperienced raters.

Patients with severe to torrential TR and a CT performed to evaluate the best treatment option (percutaneous annuloplasty vs. tricuspid transcatheter edge-to-edge repair) were enrolled in the current study. All full-cycle CTs were electrocardiogram-gated and contrast-enhanced. CTs were randomly selected and evaluated by three experienced raters, defined as radiologists with expert knowledge in cardiovascular CT analysis, and three inexperienced raters defined as cardiologists without experience in CT assessment. Measurements included tricuspid annulus area, perimeter, and diameters, as well as right ventricular length, height, and right atrial height. Assessments were performed using the automated deep learning-based analysis software heart.ai version 1 (Laralab GmbH, Munich, Germany), allowing manual adjustments if necessary. All patients provided written informed consent. All raters were informed in detail, and data were collected anonymously. The study was authorized by the institutional review board of the Charité – Universitätsmedizin Berlin, Germany (EA4/218/21). The CT analysis is described in detail elsewhere.^5^ In brief, heart.ai is a cloud-based platform with deep learning-based software using convolutional neural networks to segment the heart anatomy in major (e.g., right heart chambers) and finer structures (e.g., tricuspid valve leaflets) in all phases of the cardiac cycle (https://research.heartai-medical.com/). Multiplanar reconstruction views allow the verification of measurements and manual adjustments if necessary. The 3D and 4D models were produced for further analysis and calculation of volumes. The duration of all measurements per CT and rater was recorded except for one experienced rater due to a network-related increase in load times. One dataset per rater was collected automatically without any manual adjustments. Statistical analysis was conducted using SPSS Statistics version 28 for Windows (IBM Corporation, New York, NY, USA). Not normally distributed continuous parameters are presented as median with 25th and 75th percentiles (interquartile range [IQR]). A Mann-Whitney U test was chosen for intergroup comparisons of not normally distributed continuous parameters. The effect of a learning curve in each group was assessed using linear regression analysis with mean measurement duration as the dependent variable and the increasing number of CTs as the independent variable. Interobserver and intraobserver variability was graded by the intraclass correlation coefficient (ICC) into “excellent agreement” with an ICC >0.9 and “good agreement” with an ICC between 0.75 and 0.90. To evaluate the interobserver variability between the different groups, the mean of each parameter of one group was calculated and compared to the other group. All three raters of each group were compared to calculate the interobserver variability within one group. Moreover, Bland-Altman plots were used to visualize differences in measurements between experienced and inexperienced raters as well as the unadjusted automated analysis.

A total of 30 CTs were included in the current analysis. Experienced raters were on average faster in the complete assessment of one CT (1.8 [IQR 1.7–2.5] minutes) compared to inexperienced raters (2.6 [IQR 2.3–3.3] minutes, *p* = 0.016, Figure 1a). Both groups showed a relevant learning curve and reduced mean measurement durations from 4.5 to 1.8 minutes in the group of experienced raters (*p* = 0.006) and from 11.4 to 2.3 minutes in the group of inexperienced raters (*p* < 0.001). The mean ICC of interobserver variability was 0.957 (95% CI: 0.922–0.978) in the group of experienced raters and 0.960 (95% CI: 0.926–0.980) in the group of inexperienced raters. The ICC of interobserver variability between the groups was 0.972 (95% CI: 0.893–0.988) comparing measurements of experienced and inexperienced raters, 0.966 (95% CI: 0.865–0.986) comparing measurements of experienced raters and the unadjusted automatic CT analysis, and 0.967 (95% CI: 0.930–0.984) comparing measurements of inexperienced raters and the unadjusted automatic CT analysis. Intraobserver variability of all measurements performed by one experienced rater was 0.984 (95% CI: 0.936–0.996), and by one inexperienced rater, it was 0.994 (95% CI: 0.976–0.999). Bland-Altman plots of mid-diastolic tricuspid annulus perimeter comparing experienced and inexperienced raters as well as the unadjusted automated analysis are exemplified in Figure 1b.

**Figure 1:**
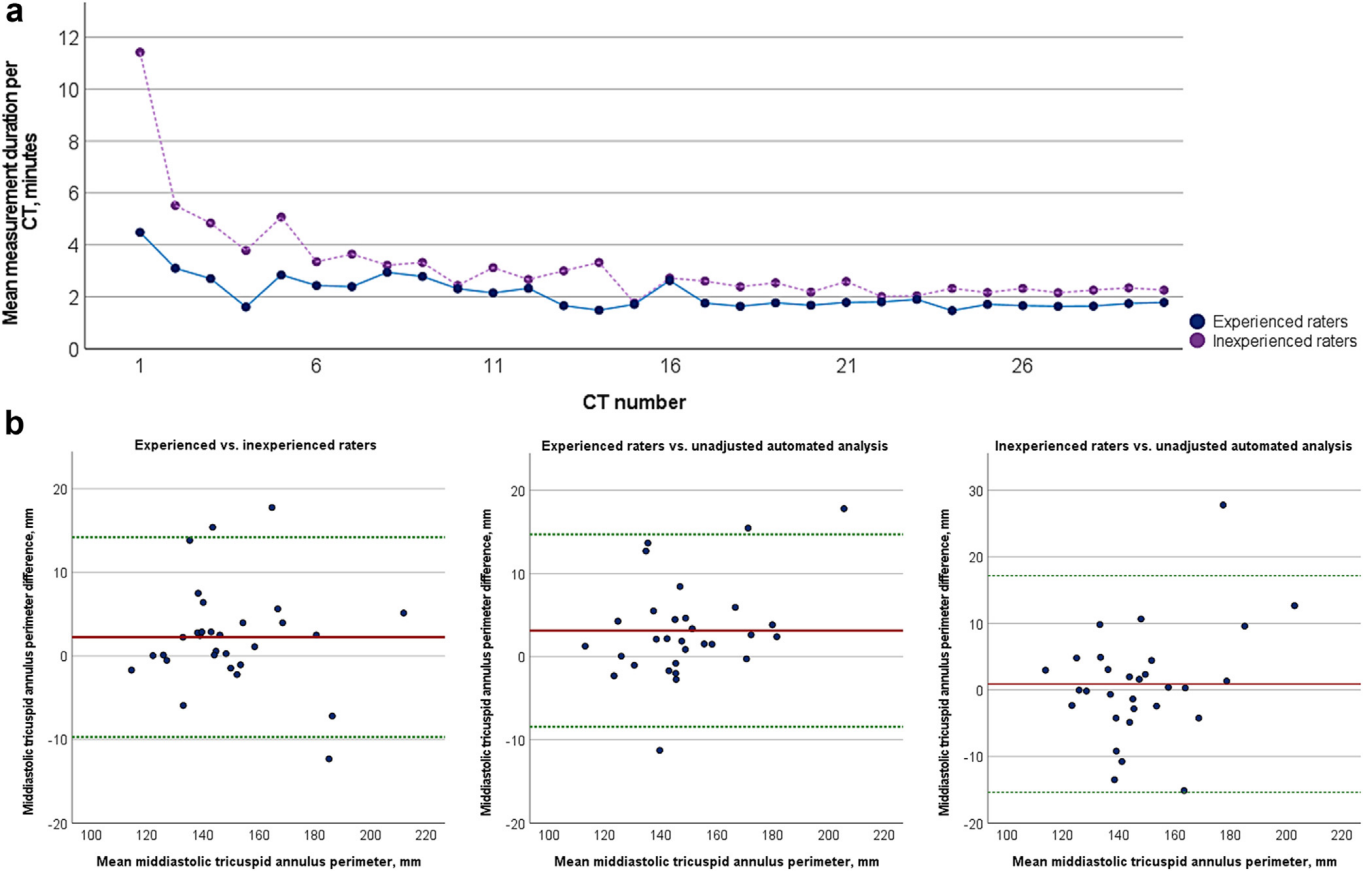
(a) Learning curve regarding mean measurement durations of experienced and inexperienced raters using an automated CT analysis. (b) Bland-Altman plots of mid-diastolic tricuspid annulus perimeter comparing experienced and inexperienced raters as well as the unadjusted automated analysis. Abbreviation: CT, computed tomography.

In summary, the present study demonstrated that a manually adjusted automated CT analysis is a precise, quick, and easy-to-use method to assess tricuspid valve and right heart morphology, irrespective of the raters’ experience. Moreover, the unadjusted automated analysis also had reliable results. Overall, the interobserver and intraobserver variability showed an excellent agreement. Differences in measurement duration between both groups decreased during the study period and may not be relevant in clinical routine.

Kirchner et al. used the automated CT analysis to evaluate right ventricular volumes and function as well as the proximity of the right coronary artery to the tricuspid annulus in patients planned for interventional TR therapy.^6^^,^^7^ The interobserver variability of unadjusted automated CT analysis and manual assessment presented an excellent agreement regarding the right ventricular volumes and a good agreement regarding the proximity of the right coronary artery, comparable to the present study.^6^^,^^7^ However, the evaluation of volumes and ejection fraction of the right ventricle required considerably more time—in detail 7.5 minutes per CT—than the analyses of areas and diameters in the current study.^6^

In recent years, various AI-based tools were developed for applications in medicine with a focus on imaging methods. These AI-based methods are efficient, reproducible, and independent from the experience of the rater resulting in an increase in accuracy for each patient.^8^ In the current study, we used deep learning-based software that allows automatic CT measurements of the right heart. Additional tools, also based on convolutional neural networks, enable a reduction in radiation and contrast fluids in CT angiography, which is another advantage of using AI.^8^ However, as AI software is trained on a specific data set from trials and clinical practice, the software can only be used for comparable patients of the training cohort.^8^ Therefore, when developing AI for daily clinical practice, minorities must be taken into account.^8^

The study has several limitations, including the small sample size of six raters and 30 CTs, as well as the network-related increase in load times of one rater. Thus, the results should be interpreted as hypothesis-generating.

To conclude, deep learning CT analysis software may assist even less imaging-experienced clinicians in planning interventional procedures in the clinical routine in the future.

## Ethics Statement

The study was approved by the institutional review board of the Charité – Universitätsmedizin Berlin, Berlin, Germany (EA4/218/21).

## Funding

Isabel Mattig is participant in the BIH Charité Clinician Scientist Program funded by the Charité – Universitätsmedizin Berlin and the Berlin Institute of Health at Charité (BIH).

## Disclosure Statement

The authors report no conflict of interest.

## References

[1] Vahanian A., Beyersdorf F., Praz F. (2022). 2021 ESC/EACTS guidelines for the management of valvular heart disease. Eur Heart J.

[2] Topilsky Y., Maltais S., Medina Inojosa J. (2019). Burden of tricuspid regurgitation in patients diagnosed in the community setting. JACC Cardiovasc Imaging.

[3] Hell M.M., Emrich T., Kreidel F. (2021). Computed tomography imaging needs for novel transcatheter tricuspid valve repair and replacement therapies. Eur Heart J Cardiovasc Imaging.

[4] Dey D., Slomka P.J., Leeson P. (2019). Artificial intelligence in cardiovascular imaging: JACC state-of-the-art review. J Am Coll Cardiol.

[5] Mattig I., Romero Dorta E., Fitch K. (2024). Predictors of residual tricuspid regurgitation after interventional therapy: an automated deep-learning CT analysis. Sci Rep.

[6] Kirchner J., Gercek M., Gesch J. (2024). Artificial intelligence-analyzed computed tomography in patients undergoing transcatheter tricuspid valve repair. Int J Cardiol.

[7] Kirchner J., Gesch J., Gercek M. (2024). Analysis of tricuspid annulus dimensions and RCA-proximity with artificial intelligence-based software for procedural planning of percutaneous tricuspid annuloplasty. J Cardiovasc Comput Tomogr.

[8] van Assen M., Razavi A.C., Whelton S.P., De Cecco C.N. (2023). Artificial intelligence in cardiac imaging: where we are and what we want. Eur Heart J.

